# Intensive care and non-invasive mechanical ventilation in kyphoscoliosis: are new perspectives still needed?

**DOI:** 10.1186/2049-6958-8-31

**Published:** 2013-05-07

**Authors:** Antonio M Esquinas, Yoshinori Matsuoka, Nalan Adıgüzel, Zuhal Karakurt

**Affiliations:** 1 Intensive Care Unit, Hospital Morales Meseguer, Avd Marques Velez, s/n, Murcia 30.008, Spain; 2 Department of Anesthesiology and Intensive Care Medicine, Saga Medical School Hospital, Saga, Japan; 3 Intensive Care Unit, Süreyyapaşa Chest Diseases and Thoracic Surgery Theaching and Research Hospital, Istanbul, Turkey

**Keywords:** Care unit, Kyphoscoliosis, Non-invasive mechanical ventilation, Intensive, Prognosis

## Abstract

Non-invasive ventilation (NIV) assists breathing and thus improves oxygenation in patients with Kyphoscoliosis. The benefits of short- and long-term intermittent nocturnal in such patients have been reported previously (improvement of vital capacity, total lung capacity, muscle strength, daytime oxygenation, exercise capacity, and pulmonary hypertension). We review this important study reporting patients with kyphoscoliosis and acute respiratory failure along with their long-term outcomes. We believe that this letter may provide important information regarding the prognosis and efficacy of NIV.

## Correspondence

Dear Editor:

Non-invasive ventilation (NIV) assists breathing and thus improves oxygenation in patients with acute respiratory failure. This effect improves outcome and quality of life in long term application [1]. Kyphoscoliosis is a common cause of pulmonary restrictive lung disease [2]. The benefits of short- and long-term intermittent nocturnal therapy have been previously reported as improvement in lung vital capacity, muscle strength, daytime oxygenation, exercise capacity, and pulmonary hypertension [2,3]. In severe stages kyphoscoliosis demonstrates impaired ventilatory mechanics and NIV appears to be the treatment of choice [3]. However, there are scarce results of prospective randomized trials to analyze NIV efficacy.

We have reviewed this original study reporting effects of NIV in kyphoscoliosis patients in acute respiratory failure and long-term outcomes [4]. We believe that it provides important information regarding the prognosis and efficacy of NIV. However, we want to add some comments regarding some particular aspects.

Firstly, contrary to previous results, the authors determined that there was no correlation between the levels of IPAP/EPAP setting applied and the levels and tendency of hypercapnia [4]. We believe that this relationship does not always exist regarding control of pCO_2_ levels and that the following factors may contribute to hypercapnia in this study such as: 1) leakage level and ventilation efficiency, 2) body mass index (BMI), 3) muscular fatigue and strength and 4) duration of NIV at home, 5) poorly controlled oxygen therapy [5]–[8].

Secondly, long-term O_2_ therapy may contribute to pCO_2_ level, significant reduction in pulmonary artery pressure (PAP) and sleep quality [2,6]. These factors were not evaluated in this study.

Thirdly, in this study the prevalence of cardiac disease remains unknown and this could be a contributing key factor in outcomes [4]. We suggest that future studies should conduct research regarding the use of transthoracic echocardiography as a routine evaluation tool for determining the response of patients with pulmonary arterial hypertension and corpulmonale, because these factors may contribute to hypoventilation and hypoxia [6,8].

We hope that our comments could improve the extrapolation of this study. In future, large prospective clinical trials will be required to evaluate this study and provide the clinical relevance of NIV in acute and long term applications.

### Reply Title: Non-invasive mechanical ventilation in the ICU is not a new perspective but varies from patient to patient with kyphoscoliosis

We thank Dr Esquinas and Dr Matsuoka for their interest and comments in helping us to improve the interpretation of our article [4].

Firstly, we agree with the contributing factors of hypercapnia [7,8]. In the Budweiser study, prior to the initiation of non-invasive mechanical ventilation (NIV), PaCO_2_ obtained from arterial blood gas, either on room air (PaCO_2_:54.5.0 ± 8.0), or normal oxygen flow (PaCO_2_:57.0 ± 7.7), was accepted as baseline in the respiratory ward [5]. However, in our study we included the baseline PaCO_2_ for the first outpatient clinic control, one month after ICU discharge, and while under NIV treatment. Therefore, the difference in PaCO_2_ in our study was low, as expected [4]. The relationship between pressure differences and PaCO_2_ differences was not correlated in stable outpatients with kyphoscoliosis and chronic respiratory failure [4]. Secondly, we did not measure pulmonary arterial pressure (PAP), and we did not perform a sleep study [4]. However, in our previous study of 34 kyphoscoliosispatients, six (24%) with chronic respiratory failure had a PAP over 40 mmHg. Surprisingly, only two (33%) who had the higher PAP had a PaO_2_/FiO_2_ less than 300 [9]. The majority of the patients (n = 28) who had abnormal PAP had hypoxemia (PaO_2_/FiO_2_ < 300) [9]. The studies on pulmonary hypertension in patients with kyphoscoliosis are very limited [3,9]. Besides hypoventilation and hypoxemia, the reasons for increased pulmonary artery pressure may involve different mechanisms. Thirdly, we recorded but did not present the cardiac co-morbidities, such as hypertension (n = 20, 32.3%), cor pulmonale (n = 14, 22.6%), and supraventricular tachycardia (n = 27, 43.5%), in our study sample.

We agree that, in future, large prospective clinical trials will be needed to assess respiratory mechanics with either spontaneous breathing, or non-invasive and invasive mechanical ventilation, with detailed cardiac function. We are willing to participate in any such study to deal with this issue.

## Abbreviations

BMI: Body mass index; EPAP: End positive airway pressure; IPAP: Inspiratory positive airway pressure; NIV: Non-invasive mechanical ventilation; PAP: Pulmonary artery pressure.

## Competing interests

Authors declare that they have no competing interests.

## Authors’ contributions

AM. E and YM carried out review analysis to prepare this manuscript. All authors read and approved the final manuscript.
